# M-GCAT: interactively and efficiently constructing large-scale multiple genome comparison frameworks in closely related species

**DOI:** 10.1186/1471-2105-7-433

**Published:** 2006-10-05

**Authors:** Todd J Treangen, Xavier Messeguer

**Affiliations:** 1 Dept. of Computer Science, Technical University of Catalonia, Barcelona, Spain

## Abstract

**Background:**

Due to recent advances in whole genome shotgun sequencing and assembly technologies, the financial cost of decoding an organism's DNA has been drastically reduced, resulting in a recent explosion of genomic sequencing projects. This increase in related genomic data will allow for in depth studies of evolution in closely related species through multiple whole genome comparisons.

**Results:**

To facilitate such comparisons, we present an interactive multiple genome comparison and alignment tool, **M-GCAT**, that can efficiently construct multiple genome comparison frameworks in closely related species. M-GCAT is able to compare and identify highly conserved regions in up to 20 closely related bacterial species in minutes on a standard computer, and as many as 90 (containing 75 cloned genomes from a set of 15 published enterobacterial genomes) in an hour. M-GCAT also incorporates a novel comparative genomics data visualization interface allowing the user to globally and locally examine and inspect the conserved regions and gene annotations.

**Conclusion:**

M-GCAT is an interactive comparative genomics tool well suited for quickly generating multiple genome comparisons frameworks and alignments among closely related species. M-GCAT is freely available for download for academic and non-commercial use at: .

## Background

Recent progress in whole genome shotgun sequencing and assembly technologies [1] has drastically reduced the cost of decoding an organism's DNA, which has resulted in a rapid increase in genomic sequencing projects. According to the Genomes OnLine Database v2.0 [2], as of August 2006 there are over 2000 active genome sequencing projects, including 413 that have already been completed and published. Of the remaining unpublished projects, there are nearly 1000 ongoing bacterial genome sequencing projects. This high concentration of bacterial genomes currently being sequenced will soon provide access to several genomes of closely related species. In fact, the *Bacillus *species alone will soon increase from 13 published genomes to 57 through active sequencing projects. Additionally, the *Yersinia *and *Salmonella *species, both will soon grow from 6 and 5 published genomes, to 22 and 23, respectively. This trend follows with *E. Coli *and *Burkholderia *species, each soon to increase from 7 to 35, and, 7 to 44 published genomes. This increase in closely related DNA sequence will allow for in depth studies of closely related species through multiple whole genome comparisons.

**Figure 1:**
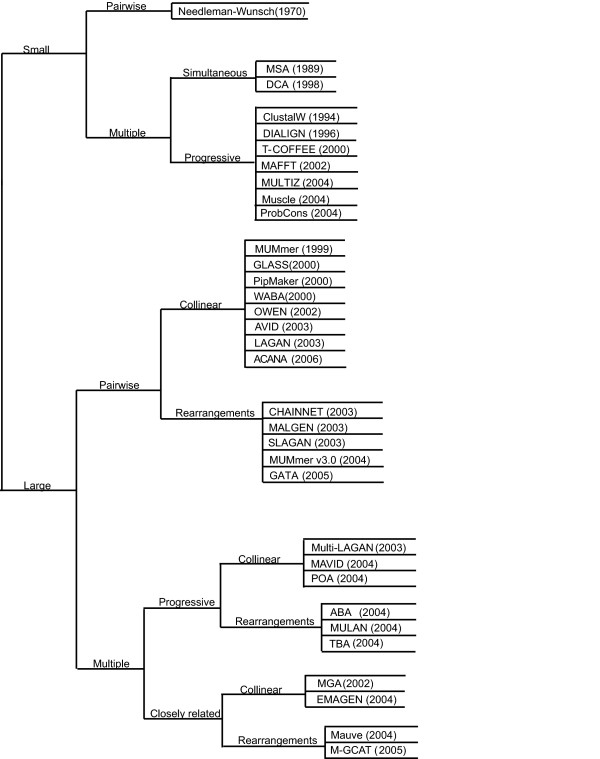
**An approximate phylogeny of genome comparison tools over the past 30 years**. Tracing the growth in related global genome comparison tools over the past 30 years.

Multiple genome comparison helps to identify biological similarities and differences in a set of genomes at the nucleotide level, identifying genomic regions that may have been conserved among several organisms. This information then can be used to make inferences about phylogeny, functional regions, and gene predictions. Figure 1 offers an approximate, and by no means complete, overview of the landscape of global comparison and alignment tools [3-19] over the last 30 years. For a more detailed review of recent advances and methodologies in comparative genomic tools see [20-23]. When designing M-GCAT, our focus was on efficient global comparisons, involving rearrangements, of multiple, closely related bacterial species. We will now describe and analyze each of these criteria in further detail.

### Comparison of large genomes

Multiple genome comparison tools need to be able to efficiently handle comparisons involving megabases of genomic sequence. However, large-scale sequence alignment does not come cheap [24]. Using traditional methods, obtaining an optimal global alignment between two sequences with more than 10,000 nt can be computationally expensive, requiring days or even months of computation time, even on well equipped computers. Thus, the first classification level in Figure 1 separates those tools able to efficiently handle small (< 10, 000 nt) or large (≥ 10, 000 nt) sequence comparisons through alignment.

### Comparison of multiple genomes

Multiple genome alignments provide for rich and sensitive comparisons that are able to identify small regions that may have been conserved or evolved among several organisms. The problem of multiple sequence alignment, however, is not in its utility but rather its complexity. Performing optimal multiple sequence alignment via dynamic programming requires *O*(*L*^*N*^) time and space complexity, where *L *is the length of the sequence and *N *the number of sequences involved in the multiple alignment [25]. This severely limits the number of genomes able to efficiently compared and aligned using such methods, which is our next classification level for the comparative genomics tools, shown in Figure 1.

### Comparison of closely related species

Due to the rapid growth in published genomic sequence, several closely related species comparisons will soon be possible. Recent progress in progressive alignment methods have allowed for thorough and accurate comparisons even among distantly related species [6,8,9,17,18,26]. While they offer high sensitivity when comparing multiple, distantly related species, such as human and fish, and avoid the reference sequence limitation [6], these tools require a quadratic number of pairwise global comparisons and can quickly become computationally expensive when comparing several large genomes. When certain assumptions can be made about the set of genomes being compared, such as the overall level of sequence similarity, alternative techniques can be used to perform simultaneous detection of matching regions in all of the genomes being compared. Methods based on multi-MUMs or multi-MEMs achieve exactly this, and are able to compare multiple, large genomes in a fraction of the time, allowing for more efficient and interactive genome comparisons. The third level of classification then separates those tools that were originally designed accurately compare and align large, multiple genomes using progressive alignment methods from those that have assumed some level of sequence conservation in the input sequences for rapid comparisons of closely related species.

### Comparisons involving rearrangements

Rearrangements can cause major variations in gene order and content among closely related organisms. Bacterial genomes often are full of rearrangements, or disorder [27,28], and large-scale inversions in bacteria were first reported in [29]. For accurate genome comparisons, it is then essential to correctly identify and track these shuffled regions to ensure an accurate global comparison of multiple bacterial genomes. As a final classification level, we distinguish between methods able to detect shuffled or rearranged similarity, such as transpositions or inversions, in multiple closely related species with large genomes from those that assume collinearity.

## Motivation and related work

Our original motivation was to design a comparative genomics tool able to keep pace with the rapid increase in the number sequenced genomes of related species and simultaneously compare 20 or more bacterial sized sequences. At the same time, we wanted to be able to interact with the generated genome comparison preview or framework. Interaction could include inspecting highly conserved regions, analyzing gene annotation, and aligning selected or all genomic regions. We now will describe a selection of existing methods related in some aspect to our original motivation, organized in three groups: (1) *Multiple genome comparison tools*, (2) *Interactive visualization tools *(3) *Multiple genome comparison tools with interactive viewers*.

### Multiple genome comparison tools

**MGA **[16] is one of the first methods capable of efficiently producing multiple whole genome alignments of closely related species. It first detects homology through searching and chaining maximal multiple exact matches, multi-MEMs [16], which are matches occurring in all genomes that cannot be extended. However, MGA was not designed to handle non-collinearity and thus it is unable to process genome rearrangements and translocations. Like MGA, **EMAGEN **[14] is able to efficiently handle multiple whole genome alignments involving collinear homology.

### Interactive visualization tools

**Enterix 2003 **[30] is a collection of three web-based visualization tools (Enteric, Menteric and Maj) for viewing previously calculated bacterial genome alignments. These tools together support a wide variety of features, including interactive alignment visualization. **GenAlyzer **[31] is an interactive tool for displaying matching substrings between two genomes. GenAlyzer can accurately display large inverted regions and match-free regions possibly caused by deletion events. **ACT **[32] allows for interactive visualization of pairwise genome comparisons generated by NCBI-BLASTN, NCBI-TBLASTX, or MUMmer [13]. ACT also displays associated annotations, and makes use of robust searching and analysis features provided by the Artemis [33] visualization tool.

### Multiple genome comparison tools with interactive viewers

**GATA **[34] is an genome comparison tool consisting of two parts, GATAligner and GATAPlotter. The GATAligner is capable pairwise sequence analysis involving rearrangements. The GATAPlotter visualization tool of segmental homology existing between the two sequences, along with rendering of corresponding gene annotation.

**Mauve **[35] is a multiple genome alignment and visualization system capable of generating comparison frameworks similar to M-GCAT. Mauve consists of a core multiple genome alignment program capable of accurately aligning multiple, large genomes while detecting lateral transfer and rearrangements, and also provides a user-friendly Java alignment viewer. Mauve uses multi-MUMs to provide its comparison frameworks, detected via a seed-and-extended hashing method, similar to that detailed in [36]. Mauve can compare more distantly related genomes than other multi-MUM based methods due to its sensitive anchors based on inexact match seeds.

## Methods

We have designed and implemented a **M**ultiple **G**enome **C**omparison and **A**lignment **T**ool, **M-GCAT**, that can efficiently and interactively compare and align large, multiple, closely related genomes involving rearrangements. Specifically, our initial goal was to efficiently establish a reliable global comparison framework to ultimately be used for alignment through efficiently detecting highly conserved regions existing in *multiple *genomes, while providing interactive alignment and inspection of conserved regions existing in all genomes.

The main distinguishing features of our method include:

### Efficient construction of comparison frameworks in multiple species

Figure 4 depicts what we have defined as an *comparison framework: *an interactive picture of the most similar regions between all genomes based on the set of detected multi-MUMs. M-GCAT relies on a compressed suffix tree string searching algorithm to identify the multi-MUMs. This algorithm has linear time complexity with respect to the combined length of all genomes, and the current implementation uses approximately 24 bytes per nucleotide contained in the smallest sequence, and two bytes per character for the remaining sequences. One of the main advantages of our compressed suffix tree search algorithm is its speed and simplicity when comparing multiple genomes. Further details of our multi-MUM searching algorithm can be found in [37]. This approach allows us to efficiently handle multiple comparisons involving 20 or more genomes. However, as previously described in [14,35], genome comparisons based around unique matches will encounter difficulty with repetitive regions, especially large segmental duplications. This will often result in the algorithm dedicating a large amount of time searching for additional, smaller matches during recursive anchoring in hopes of identifying all or part of previously unidentified regions. Also, generally speaking, multi-MUMs require high sequence conservation to generate reliable comparisons, and even so can result in low sequence coverage.

Our algorithm for generating comparison frameworks by clustering multi-MUMs involves the following four sequential steps: *(1) Anchoring, (2) Recursive Anchoring, (3) Filtering *and *(4) Clustering*.

#### 1. Anchoring

To be able to efficiently align entire genomes it is necessary to try to limit the dynamic programming search space through heuristics. Anchoring is one such heuristic, can be used to establish a framework of conserved sequence among all sequences being compared. Anchoring has been used in several global alignment tools, such as [7,9,13,38]. M-GCAT anchors are established by finding all statistically significant Maximal Unique Matchings (MUMs [12]) among all genomes via an efficient multi-MUM searching algorithm. The parameter **Min Anchor length **inputs the minimum allowable size for the initial set of multi-MUM anchors found among all genomes. The default value is log_2_(length(S_1_)), where *S*_1 _is the reference sequence.

#### 2. Recursive anchoring

This step involves searching for significant multi-MUMs between established anchors common in all genomes. The goal is to scour the genomes for as much matching genomic sequence as possible by searching the regions that lie between anchors for additional shorter multi-MUMs and thus creating new regions small enough to be efficiently aligned. Two parameters limit this step: the **Min MUM length **and **the q value**. The first determines the minimum allowable length for new multi-MUMs found between the anchors during the recursive anchoring process and the second determines the minimum allowable length in nucleotides of a searchable sequence region *R*_*n*_. As searchable sequence regions become smaller and smaller, so should this value. The default value is 1.3 * log_2_(length(R_*n*_)), where *R*_*n *_is a searchable sequence region in sequence *n *and 1.3 is a coefficient that should be decreased when dealing with more distantly related species. Both the **Min MUM length **and **Min Anchor length **equations can be manually edited by the user and so can be configured to also decrease with respect to the number of the genomes involved in the comparison.

#### 3. Filtering

In order to remove any spurious matches found in the previous step we employ a filtering step. Filtering attempts to remove all noise generated by the recursive anchoring process by identifying all multi-MUMs with length that is less than a **Random MUM length **parameter and that induce spurious breakpoints in homology. All multi-MUMs less than or equal to this length that are also breakpoints in conserved sequence collinearity are considered to be random will be removed in the Filtering process. The default value is 0. Using this filtering technique, the maximum set of collinear multi-MUMs can be calculated by setting the Random MUM value to the length of the reference sequence. Then, all multi-MUMs that do not coincide with the main collinear structure of the homology will be discarded.

#### 4. Clustering

To organize all of the highly conserved regions found in the previous steps, we group collinear multi-MUMs into multi-MUM clusters (see Figure 3). Before clustering the multi-MUMs, we remove all overlaps of all of the matches so that no nucleotide is contained in more than one multi-MUM Cluster. There is no maximum number of multi-MUMs that can be stored in a given Cluster, and any non-random multi-MUM that is not collinear to any other multi-MUM will form its own Cluster. The parameter d is the maximum allowable distance, in nucleotides, between any two adjacent multi-MUMs in a cluster. Increasing this value will generally increase the alignment time, and decreasing this value will generally decrease the percentage of the genomes that will be aligned. Setting **d **to the length of the longest sequence will minimize the number of Clusters, separating regions in the multiple genome comparison strictly by breakpoints in collinearity. The default value is l000 nt.

Each multi-MUM is compared to each other to check the collinearity and distance constraint. First, when clustering the multi-MUMs, we start with the leftmost MUM, ordered with respect to its position in the reference sequence, and then proceed to the right considering only the multi-MUMs within the distance d, and that is collinear to the previous multi-MUM. Resultantly, each multi-MUM in a Cluster is collinear to its left and right neighbor and within d nucleotides.

Clusters of multi-MUMs aid in understanding the global homology structure between the candidate genomes and facilitates the automatic computation of gapped global alignments across the entire genomes. Furthermore, multi-MUM Clusters are designed to indicate all related regions and serve as visual cues for quickly identifying large-scale genome rearrangements, such as inversions.

### Interactive and visual comparison environment

M-GCAT offers the ability to interactively inspect and align any conserved region among multiple genomes by simply highlighting and selecting it with the mouse. It provides a full-featured graphical user interface, with interactive visualization of matching regions in all genomes that is similar in spirit to ACT [32], GATA [34], and GenAlyzer [31].

There are five workspaces, each equipped with an array of configurable features and options, designed to provide a distinct working environment based on each interactive task. The main workspace is the **Gene viewer workspace **in which any selected region can be aligned, displayed with gene information or sent as a NCBI-BLASTN web query with the results incorporated inside of the user interface. These features allow the user to manually inspect and verify the various conserved regions that have been detected by M-GCAT. The gene information is provided by the PTT files of NCBI. As the PTT files are simply flat text files, revision of existing annotations and addition of new annotations is easily accomplished. All genes extracted from a genome annotation are incorporated into the multi-MUM Clusters of highly similar regions. Then, visually all genes can be navigated and viewed region by region (see figure 3), which can prove useful when trying to identify islands of conserved similarities, gene duplications and insertions, or for viewing patterns of proximity and function of genes. To date, all bacterial genomes available on the NCBI ftp site have a corresponding PTT file. Detailed descriptions of the five available workspaces follows.

**• Gene viewer workspace **(Figure 3): this is the default workspace inside the graphical user interface of M-GCAT. The topmost window displays the multi-MUM clusters found between these two sequences, which is the global framework that will be used to build the alignment. The window immediately below shows information relevant to the highlighted MUM cluster (light green). Any region can be aligned using MUSCLE [39], and when finished the information is stored for future reference. The quality of the alignment is scored and displayed visually, ranking from low identity (light yellow) to high identity (dark red). The bottommost window is the gene map, and is derived from a PTT file that corresponds to each sequence. Individual genes can be selected and any relevant information for a selected gene is displayed in the window adjacent to the gene map window. The example provided in Figure was generated using a set of four *Yersinia *genomes, further details of this comparison can be found on the M-GCAT website under the Experiments section.

**• MUM Workspace**: Contains two windows used for displaying a visual representation of multi-MUMs found among all sequences, along with any relevant information. Each multi-MUM can be selected to view its length, start and end positions in the bottom window.

**• Cluster Workspace**: Contains two windows used for displaying all of the multi-MUM clusters found among all sequences, along with any relevant information. Each cluster can be selected to view its length, start and end positions in the bottom window. Additionally, the clusters can be lined up and traced with the mouse movement.

**• MUM & Cluster Workspace**: Joins all of the information in the MUM Workspace and Cluster Workspace into one. In this mode, the zoom and movement can be put in sync so that the relationships between the multi-MUMs, multi-MUM clusters, and the *d *value can be easily studied.

**• Alignment viewer Workspace**: The Alignment viewer Workspace joins the Cluster Workspace with an additional window containing the alignment results from the resulting MUSCLE alignment if the selected cluster has been aligned. If it has not been previously aligned, a new alignment can be performed by selecting *Align *=> *Align selected region *from the Main Menu Bar.

### Genome sequence partitioning

Often times the smallest sequence involved the comparison is millions of nucleotides in size, and can resultantly require more than 1 GB of system memory to perform the comparison. To limit memory usage and allow standard desktop computers with less than 1 GB memory to compare large genomes, we have devised a partitioning scheme for our compressed suffix tree based multi-MUM searching method such that we can partition the smallest sequence into subsequences in exchange for an increase in runtime. The increase in runtime results from the additional compressed suffix trees that are created, 1 per each partition, in conjunction with the time required to merge the results from each partition into a complete set of multi-MUMs across all of the partitions. The parameter **P **determines the length of the parts. The default value is 10,000,000 nt. A comparison involving a sequence of 20,000,000 nt would then require approximately 50% less memory, but would roughly imply a 200% increase in runtime.

### Sensitive and configurable homology detection

M-GCAT will group all collinear multi-MUMs into clusters based on a distance parameter, d, which stipulates that only multi-MUMs that are at most separated by d nucleotides can be grouped inside of the same multi-MUM cluster. This subtle requirement allows the user to either highlight only the highly conserved regions in all genomes by setting the d value near 0, or to show the maximal global comparison framework separated by breakpoints in collinearity by setting the d value to the length of the largest sequence. This feature also allows for interactive tailoring of the framework before spending several hours running a full alignment.

## Implementation

M-GCAT was implemented in the C++ and Python programming languages. This software has been compiled and tested on Windows, Linux, MacOS X, and Solaris. When performing large genome comparisons it is necessary to have at least 512 MB RAM available, and 1024 MB is recommended. M-GCAT consists of two components, (1) The core genome comparison program written in C++, mgcat, and (2) an interactive viewer and alignment tool written in python, viewer.py. Both components are required to achieve full functionality of the software. For supported Windows versions (98, 2000, XP) no additional software or libraries are required. For non-Window platforms, it is necessary to install Python version 2.3 or higher along with Tcl/Tk 8.3 or higher. Additionally, the python script, shuffleGenome.py, used to shuffle the genomes and introduce large-scale rearrangements is available for download on the M-GCAT website.

### Program input

**Figure 2:**
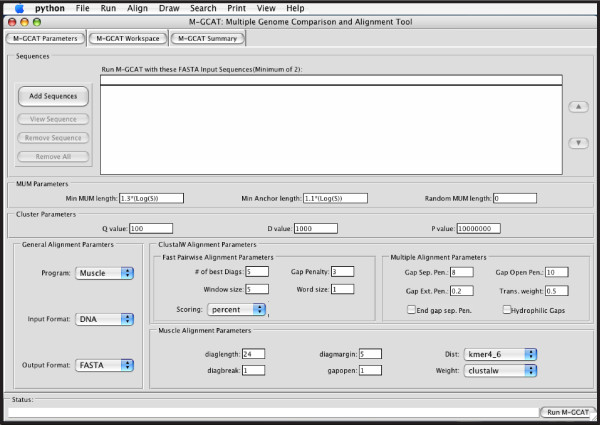
**The M-GCAT parameter page**. The M-GCAT user interface parameter page. When M-GCAT is started, this is displayed to allow the user to select the input sequences, modify the main parameters, and load previously saved M-GCAT comparisons.

Figure 2 shows the M-GCAT Parameter page, where it is possible to select input sequences, configure the main parameters, and load previously saved M-GCAT comparisons. For starting a new comparison, M-GCAT accepts FASTA formatted DNA sequences. The memory required for each comparison will depend on the length of the reference sequence. Assuming the reference sequence is the smallest in the set to be compared, this will limit the comparisons as follows: 5–10 Mb reference sequence requires approximately 512 MB RAM, 10–20 Mb reference sequence approx. 1024 MB RAM, and so on. To get around this limitation we allow the reference sequence to be partitioned into smaller parts, allowing comparisons involving sequences twice as long while requiring 50% less system memory. Also, it is necessary to provide a corresponding PTT file in the same directory as the FASTA sequence file, with the same name (for example, *sequence.fna *&*sequence.ptt*). in order to properly view genome annotations.

### Running M-GCAT

After configuring the parameters (see Additional file 3), M-GCAT can be started through the python GUI by selecting **Run M-GCAT**. When python is not available or when running large comparisons, M-GCAT can be called from the command line as follows: mgcat *mgcat.ini*. All of the parameters can be set inside of a specified configuration file, *mgcat.ini*. The program upon completion writes all output to the ./*output *directory.

### Output files

M-GCAT generates four output files after each successful comparison, and an additional alignment output file after each successful MUSCLE alignment. Specifically, each file contains:

• **ANCHORS**: All multi-MUM Anchors found during Anchoring phase.

• **MUMS**: All multi-MUMs found during the Anchoring AND Recursive anchoring phase

• **MGCAT**: All of the multi-MUM Clusters, which contain multi-MUMs and the regions between any two collinear multi-MUMs.

• **LOG**: This file contains a summary of results for a successfully completed genome comparison. To view the LOG file, select the 'M-GCAT Summary' Tab. This will list information relevant to the genomes such as size and name, as well as other useful information.

• **ALIGN**: M-GCAT alignment data. This file contains a sequential list of partial alignments. The alignment output can also be saved in two additional formats, **MLN **[35] (Mauve alignment format) and **XMFA **[10] (Shuffle-LAGAN alignment format).

## Results

Table 1 provides a survey of M-GCAT's performance on a set of 20 independent sequence comparisons each involving a selected set of closely related species. The efficiency of our approach stands out when attempting to compare several genomes at a time, as in comparison $17 and $21. For a detailed list of all genomes involved in the experiments see Additional file 2. Most of the multi-MUM anchors in the were generated in a few seconds, comparison frameworks in less than 5 minutes, and required less than 1 GB of system memory. This includes the integrated detection of rearrangements, such as inversions, allowing M-GCAT to rapidly locate large-scale inversions existing in all of the genomes without requiring a quadratic number of comparisons.

**Table 1:** A summary of experimental results for 23 distinct sets of sequences

#	Sequence set	Size	A MathType@MTEF@5@5@+=feaafiart1ev1aaatCvAUfKttLearuWrP9MDH5MBPbIqV92AaeXatLxBI9gBamrtHrhAL1wy0L2yHvtyaeHbnfgDOvwBHrxAJfwnaebbnrfifHhDYfgasaacH8akY=wiFfYdH8Gipec8Eeeu0xXdbba9frFj0=OqFfea0dXdd9vqai=hGuQ8kuc9pgc9s8qqaq=dirpe0xb9q8qiLsFr0=vr0=vr0dc8meaabaqaciaacaGaaeqabaWaaeGaeaaakeaaimaacqWFaeFqaaa@3821@_*size*_	A MathType@MTEF@5@5@+=feaafiart1ev1aaatCvAUfKttLearuWrP9MDH5MBPbIqV92AaeXatLxBI9gBamrtHrhAL1wy0L2yHvtyaeHbnfgDOvwBHrxAJfwnaebbnrfifHhDYfgasaacH8akY=wiFfYdH8Gipec8Eeeu0xXdbba9frFj0=OqFfea0dXdd9vqai=hGuQ8kuc9pgc9s8qqaq=dirpe0xb9q8qiLsFr0=vr0=vr0dc8meaabaqaciaacaGaaeqabaWaaeGaeaaakeaaimaacqWFaeFqaaa@3821@	ℳ MathType@MTEF@5@5@+=feaafiart1ev1aaatCvAUfKttLearuWrP9MDH5MBPbIqV92AaeXatLxBI9gBamrtHrhAL1wy0L2yHvtyaeHbnfgDOvwBHrxAJfwnaebbnrfifHhDYfgasaacH8akY=wiFfYdH8Gipec8Eeeu0xXdbba9frFj0=OqFfea0dXdd9vqai=hGuQ8kuc9pgc9s8qqaq=dirpe0xb9q8qiLsFr0=vr0=vr0dc8meaabaqaciaacaGaaeqabaWaaeGaeaaakeaaimaacqWFZestaaa@3790@	C MathType@MTEF@5@5@+=feaafiart1ev1aaatCvAUfKttLearuWrP9MDH5MBPbIqV92AaeXatLxBI9gBamrtHrhAL1wy0L2yHvtyaeHbnfgDOvwBHrxAJfwnaebbnrfifHhDYfgasaacH8akY=wiFfYdH8Gipec8Eeeu0xXdbba9frFj0=OqFfea0dXdd9vqai=hGuQ8kuc9pgc9s8qqaq=dirpe0xb9q8qiLsFr0=vr0=vr0dc8meaabaqaciaacaGaaeqabaWaaeGaeaaakeaaimaacqWFce=qaaa@3825@	*t*_*anchor*_	*t*_*mum*_	*t*_*total*_	*Mem*	*Cov*.
1	*Mycoplasma *2	1.5	22	264	6325	649	2s	5s	8s	52	72.8
2	*Pyrococcus *2	3.5	23	1159	3229	484	6s	3s	9s	153	62.5
3	*Salmonella *2	9.6	27	470	516	39	16s	1s	17s	419	98.9
4	*Listeria *3	8.7	24	13101	45940	722	16s	114s	143s	283	94.3
5	*X. Campestris *3	15.3	27	15843	37702	2441	25s	151s	181s	487	74.8
6	P. *Syringae *3	18.4	27	11232	39753	1527	35s	252s	294s	573	72.8
7	*C. Pneumoniae *4	4.9	21	770	0	7	6s	0s	6s	156	98.5
8	*Yersinia *4	21.4	25	14049	6	400	24s	1s	25s	488	94.0
9	*Shigella 5*	23.1	23	37596	1285	564	32s	2s	38s	548	76.7
10	*Salmonella 5*	23.8	23	46336	983	328	35s	1s	39s	567	93.1
11	*E. Coli 5*	25.3	23	47221	5543	704	38s	9s	57s	553	84.2
12	*Streptococcus 7*	13.1	21	15446	84	121	17s	1s	18s	258	88.3
13	*Staphylococcus 7*	19.6	24	23216	132	260	24s	2s	26s	390	92.6
14	*Bacillus 7*	36.8	25	27731	4149	468	54s	7s	62s	713	93.2
15	*Entero *10 (9&11)	48.4	23	39979	3753	418	63s	12s	78s	740	73.3
16	*Entero *15 (9&10&11)	72.2	23	5802	8136	1218	95s	84s	181s	991	54.9
17	*Entero *19 (8&9&10&11)	93.6	18	1132	637	907	161s	99s	261s	1174	15.6
18	Bacilli 14 (12&13)	22.7	19	251	3801	2721	43s	41s	93s	414	14.3
19	Bacilli 14 (13&14)	56.4	19	431	5250	2718	79s	100s	185s	654	26.3
20	*Bacilli *21 (12&13&14)	62.3	15	597	1691	2045	100s	54s	155s	638	4.1

A selection of results for 20 independent sets of closely related sequence comparisons conducted with M-GCAT. Size and Memory usage are listed in megabytes (MB). All experiments were performed and running times (cpu time) measured on a 2 GHZ Pentium processor, with 2 GB of main memory, running Windows XP Professional. Size is the total size (MB) of the set of sequences. A
 MathType@MTEF@5@5@+=feaafiart1ev1aaatCvAUfKttLearuWrP9MDH5MBPbIqV92AaeXatLxBI9gBamrtHrhAL1wy0L2yHvtyaeHbnfgDOvwBHrxAJfwnaebbnrfifHhDYfgasaacH8akY=wiFfYdH8Gipec8Eeeu0xXdbba9frFj0=OqFfea0dXdd9vqai=hGuQ8kuc9pgc9s8qqaq=dirpe0xb9q8qiLsFr0=vr0=vr0dc8meaabaqaciaacaGaaeqabaWaaeGaeaaakeaaimaacqWFaeFqaaa@3821@ is the number of multi-MUM Anchors found, A
 MathType@MTEF@5@5@+=feaafiart1ev1aaatCvAUfKttLearuWrP9MDH5MBPbIqV92AaeXatLxBI9gBamrtHrhAL1wy0L2yHvtyaeHbnfgDOvwBHrxAJfwnaebbnrfifHhDYfgasaacH8akY=wiFfYdH8Gipec8Eeeu0xXdbba9frFj0=OqFfea0dXdd9vqai=hGuQ8kuc9pgc9s8qqaq=dirpe0xb9q8qiLsFr0=vr0=vr0dc8meaabaqaciaacaGaaeqabaWaaeGaeaaakeaaimaacqWFaeFqaaa@3821@_*size *_is the configured minimum size of multi-MUM Anchors, ℳ
 MathType@MTEF@5@5@+=feaafiart1ev1aaatCvAUfKttLearuWrP9MDH5MBPbIqV92AaeXatLxBI9gBamrtHrhAL1wy0L2yHvtyaeHbnfgDOvwBHrxAJfwnaebbnrfifHhDYfgasaacH8akY=wiFfYdH8Gipec8Eeeu0xXdbba9frFj0=OqFfea0dXdd9vqai=hGuQ8kuc9pgc9s8qqaq=dirpe0xb9q8qiLsFr0=vr0=vr0dc8meaabaqaciaacaGaaeqabaWaaeGaeaaakeaaimaacqWFZestaaa@3790@ is number of multi-MUMs found, C
 MathType@MTEF@5@5@+=feaafiart1ev1aaatCvAUfKttLearuWrP9MDH5MBPbIqV92AaeXatLxBI9gBamrtHrhAL1wy0L2yHvtyaeHbnfgDOvwBHrxAJfwnaebbnrfifHhDYfgasaacH8akY=wiFfYdH8Gipec8Eeeu0xXdbba9frFj0=OqFfea0dXdd9vqai=hGuQ8kuc9pgc9s8qqaq=dirpe0xb9q8qiLsFr0=vr0=vr0dc8meaabaqaciaacaGaaeqabaWaaeGaeaaakeaaimaacqWFce=qaaa@3825@ is the number of multi-MUM clusters. *t*_*anchor *_is the time needed to find the set of multi-MUM anchors, *t*_*mum *_is the time needed to find the initial set of multi-MUMS, and *t*_*total *_the time required to perform entire comparison. *Mem *is peak usage of system memory (MB), and *Cov*. is the percentage of each sequence that was aligned. The percentage that was not aligned corresponds to regions where no multi-MUMs were found. A *p *value of 10,000,000 and *q *value of 100 was used for all experiments. The *d *value was set to the length of the longest sequence in each example to emphasize the global alignment framework. For a complete listing of the sequences used in these comparisons refer to Additional file 2.

### Multi-MUM performance

We have compared the performance of our algorithm against two similar multiple genome comparison tools, MGA [16] and EMAGEN [14]. Both rely on suffix arrays for efficient multi-MUM/MEM search in large genomes. However, both MGA and EMAGEN assume collinearity and thus are not well suited for detecting large-scale rearrangements, such as transpositions or inversions. That said, using the results presented in [14], we have conducted a comparison of their sensitivity with our approach. As the results are specifically for the longest increasing subsequence of multi-MUMs, or LIS-MUMs, our algorithm had to be adapted before performing the analysis to filter out all MUMs not appearing in the longest collinear chain of matches. Additionally, we ran the implementation of our method on a comparable Sun workstation. Our results we generated on a Sun Ultra-250 Spare II 400 Mhz computer, with 512 MB RAM. As reported in [14], the remaining results in Table 1 were generated on a Sun Blade 1000 workstation (UltraSPARC III 750 Mhz) with 1 gigabyte of RAM.

In general, the performance of our method for the data employed with respect to MUM search time is significantly faster that MGA, and is comparable to EMAGEN. At the same time, the sensitivity of our multi-MUM detection is near identical to that obtained by MGA. However, M-GCAT achieves a significant improvement in efficiency of suffix structure construction. We attribute this to our streaming method that requires only the smallest genome in the comparison to be indexed.

Furthermore, it was reported in [14] that: "The only obvious break between bp positions of 1 and 2 millions indicates that the major difference among three strains is located in this region." We decided to extend our analysis to include rearrangements to see if M-GCAT could account for this discrepancy, as this break between the genomes has been described in [40] to be a large-scale inversion between two of the genomes involved in the comparison, *E. Coli K12 *and *E. Coli O157:H7*. The inversion was reported to be 422 kb in length, and was detected by M-GCAT evident by the total match coverage increase from approximately 3.6 mb to 4.0 mb. As previously mentioned, inversions of this type in bacteria are not a novelty and were first reported in [29]. In fact, bacterial genomes are often full of rearrangements, or disorder [27,28], making it essential to correctly identify these regions to ensure an accurate global comparison of multiple bacterial genomes. This is exemplified in the second example involving the four strains of *Streptococcus*, which contains a 1 megabase X-alignment [29], or symmetric inversion, accounting for the large discrepancy in the total length of the LIS multi-MUMs.

### Genome comparison framework efficiency

To better evaluate the efficiency of generating genome comparison frameworks with M-GCAT, we have compared it to the Mauve whole genome alignment system. Mauve was one of the first methods able efficiently detect rearrangements in multiple whole genomes via multi-MUMs, and so we have compared our method to Mauve's performance on a set of closely related genomes. While we realize the inherent pitfalls with such comparisons, we felt it was a reasonable gauge of the efficiency of our approach. Thus, to evaluate the efficiency we have performed a series of 8 experiments and compared the performance based on cpu time. The experiment involves 8 sets of closely related enterobacterial genomes, ranging in size from 3 to 21. The sequences used from 3 to 15 are consistent with sequence set #16 from Table 1. The remaining 6 genomes were generated by shuffling 6 of the 15 published genomes with our genome shuffling script *shuffleGenome.py *to introduce new cloned bacterial genomes, each containing five new large scale rearrangements of 50,000 nt.

For this experiment the parameters were configured as follows. The Mauve *Full Alignment *option was disabled, and a default mer size of 23 was used. The M-GCAT **Min Anchor Length **parameter was set to 23, the *d *value to the length of the largest genome to roughly emulate the behavior of the Locally Collinear Blocks, and the remaining parameters were configured using appropriate values for the context of each experiment. For example, **Min MUM Length **was set to the default value of 1.3 * log_2_(length(*R*_*n*_)) for experiments involving 3 & 6 genomes, and reduced thereafter up until the 21 genome experiment where it was configured to be 0.7 * log_2_(length(*R*_*n*_)). Similarly, the **Random MUM Length **was set higher for smaller test cases, and gradually decreased for each successively larger experiment.

Table 4 compares the comparison frameworks generated by both methods for the first 3 cases. The number of Clusters & LCBs for these examples are close to the same, Mauve tends to cover the same regions produced with M-GCAT, and often times more genomic sequence, with its LCB frameworks. We believe the increased coverage can be explained by the more sensitive inexact match seeds employed by Mauve. For all cases, M-GCAT consistently required less time than Mauve, and Mauve begins to rapidly increase in runtime after 15 genomes, notably increasing from 93 minutes to 13 hours of cpu time for 18 to 21 genomes, respectively. While both methods require less than 1 GB for the comparisons involving up to 15 enterobacterial genomes, M-GCAT's memory usage tends to exceed that of Mauve, except for the 18 genome example (see Figure 5(a)). We attribute this to M-GCAT's initial memory overhead to generate and store the compressed suffix tree, which stabilizes after the first few sets and then requires a fixed amount of memory per additional genome added (see Figure 5(b)).

### Validating the reliability the comparison frameworks

While our approach focuses on efficiently providing comparison frameworks for large sets of closely related genomes, we also need to ensure that we are generating *reliable *frameworks. The reliability will directly affect the multiple genome alignments prepared with M-GCAT and other methods, and if we are correctly identifying orthologous genes in the genomes we can be more confident that we will generate accurate multiple genome alignments. Thus, to validate the reliability of our results, we have tested the accuracy of the generated comparison frameworks on a selected set of experiments in Table 1.

We have measured the accuracy by calculating the percentage of the known orthologous genes that are located in the same multi-MUM cluster. We use the COG [41] identifier to determine if two or more genes from distinct genomes are orthologous. If the COG identifier was not available for a gene, we then used the gene name. If neither COG or gene name is available for gene, it was classified as unknown. To limit the number of unknowns, so we have chosen a set of well annotated genomes. The results of this test of these 5 sets are detailed in Table 2. For all cases we achieve relatively high accuracy, and up to 94.2% in the *Entero *10 example. Also, the accuracy appears to increase with the number of closely related genomes involved in the comparison. This is true as multiple comparisons help to filter out the spurious multi-MUMs and cause less orthologous genes to be *missed*. Most of the *missed *orthologs can arise with ambiguous orthology due to repetitive regions and gene duplication. Another reason for lowered accuracy could be due to the sensitivity of our exact match method, while fast, it cannot account for small changes to insertions and deletions to orthologous regions. We have marked this as a opportunity for future work.

**Table 2:** Verifying reliability of selected Alignment frameworks

#	Sequence set	C MathType@MTEF@5@5@+=feaafiart1ev1aaatCvAUfKttLearuWrP9MDH5MBPbIqV92AaeXatLxBI9gBamrtHrhAL1wy0L2yHvtyaeHbnfgDOvwBHrxAJfwnaebbnrfifHhDYfgasaacH8akY=wiFfYdH8Gipec8Eeeu0xXdbba9frFj0=OqFfea0dXdd9vqai=hGuQ8kuc9pgc9s8qqaq=dirpe0xb9q8qiLsFr0=vr0=vr0dc8meaabaqaciaacaGaaeqabaWaaeGaeaaakeaaimaacqWFce=qaaa@3825@	*Identified*	*Missed*	*Known*	*Unknown*	*Total*	*Accuracy*
1	*Mycoplasma *2	649	1188	244	1432	576	2008	82.0%
2	*Pyrococcus *2	484	1971	585	2556	675	3231	77.0%
13	*Salmonella 5*	328	12108	3823	15931	4953	20884	76.0%
18	*Entero *10	418	28428	1757	30185	5375	35560	94.2%
19	*Entero *15	1218	42617	2883	45550	7291	52791	93.7%

Testing the reliability of five of the alignment frameworks generated in Table 1. C
 MathType@MTEF@5@5@+=feaafiart1ev1aaatCvAUfKttLearuWrP9MDH5MBPbIqV92AaeXatLxBI9gBamrtHrhAL1wy0L2yHvtyaeHbnfgDOvwBHrxAJfwnaebbnrfifHhDYfgasaacH8akY=wiFfYdH8Gipec8Eeeu0xXdbba9frFj0=OqFfea0dXdd9vqai=hGuQ8kuc9pgc9s8qqaq=dirpe0xb9q8qiLsFr0=vr0=vr0dc8meaabaqaciaacaGaaeqabaWaaeGaeaaakeaaimaacqWFce=qaaa@3825@ is the number of multi-MUM clusters analyzed for orthologs, *Identified *is the total number of genes with one or more identified orthologs in its corresponding multi-MUM cluster, *Missed *the total number of proteins in multi-MUM clusters with no orthologs, *Unknown *the total number of genes that have yet to be fully classified, *Total *the total number of genes in all of the multi-MUM clusters in all genomes, and *Accuracy *the number of *Identified *orthologs divided by the total *Known *(*Identified *+ *Missed*).

### Verifying scalability

Finally, to test the scalability of our method with respect to the number of genomes, we have generated a multiple genome comparison framework for 90 bacterial genomes. To create such a large set of closely related sequences, we took the 15 published enterobacterial genomes of *E. Coli, Shigella*, and *Salmonella*, then we shuffled each genome 5 times in order to introduce large scale rearrangements of 50,000 nt in length which could be considered consistent with those found in the first 6 sets. The five rearrangements per genome introduced included transpositions, inversions, and inverted transpositions. Then, we performed a full comparison of all of the 90 related genomes and recorded the cpu time and memory usage. The multiple genome comparison framework for this large set of closely related genomes involving rearrangements was constructed in approximately 1 hour, and consumed 6.5 gigabytes of memory. The comparison was run on a Sun Microsystems Sun Fire V440 with a sparcv9 1062 Mhz cpu and 8 GB system memory, and all of the output files, along with a image of the genome comparison framework, is available as Additional file 1.

## Discussion and conclusion

We have presented an interactive environment for efficient genome comparisons, M-GCAT, which compares favorably to related existing methods. We have provided four experiments to validate this claim by testing the *efficiency, reliability *and *scalability *of our method. In general, our multi-MUM based genome comparison frameworks achieve good results when comparing closely related genomes. However, our multi-MUM based method has several limitations, such as the reference sequence limitation [6], problems with large segmental duplications, reduced anchor sensitivity [35], current inability to handle inexact matches, and relatively large memory overhead in comparison to other suffix structures [13,16]. As future work we hope to address these limitations, as well as to better extend our current method from generating reliable genome comparison frameworks to global alignments. This would allow us to better verify and test our multiple genome alignments with existing related methods. That said, our multiple comparison frameworks can currently be used directly to generate whole genome alignments, as well as a pre-processing step with several existing methods to improve runtime for otherwise computationally limiting comparisons. In addition to this, we plan to expand M-GCAT's input capabilities to support common formats so that the python viewer itself can be readily used to provide an interactive and visual environment for many existing multiple genome comparison tools that lack an interactive visualization environment.

## Availability and requirements

**Project Name: **M-GCAT

**Project website: **

**Operating system: **Linux, Mac OS X, Solaris, and Windows

**Programming language: **C++ and Python

**License: **M-GCAT is freely available for download for academic and non-commercial use.

## Abbreviations

**MUM: **Maximal Unique Match

**multi-MUM: **Maximal Unique Match occurring in multiple sequences

**MEM: **Maximal Exact Match

**PTT: **Protein Table File

**PYC: **Python Compiled file

**GUI: **Graphical User Interface

**COG: **Cluster of Orthologous Groups

## Authors' contributions

TT participated in the design of the algorithms, implemented the current version in *C++ *and Python, tested the software, created the project website, and drafted the manuscript. XM directed the project, participated in the design of the algorithms, assisted in the implementation of the software, and assisted in writing the manuscript. Both authors have approved the final manuscript.

**Figure 3:**
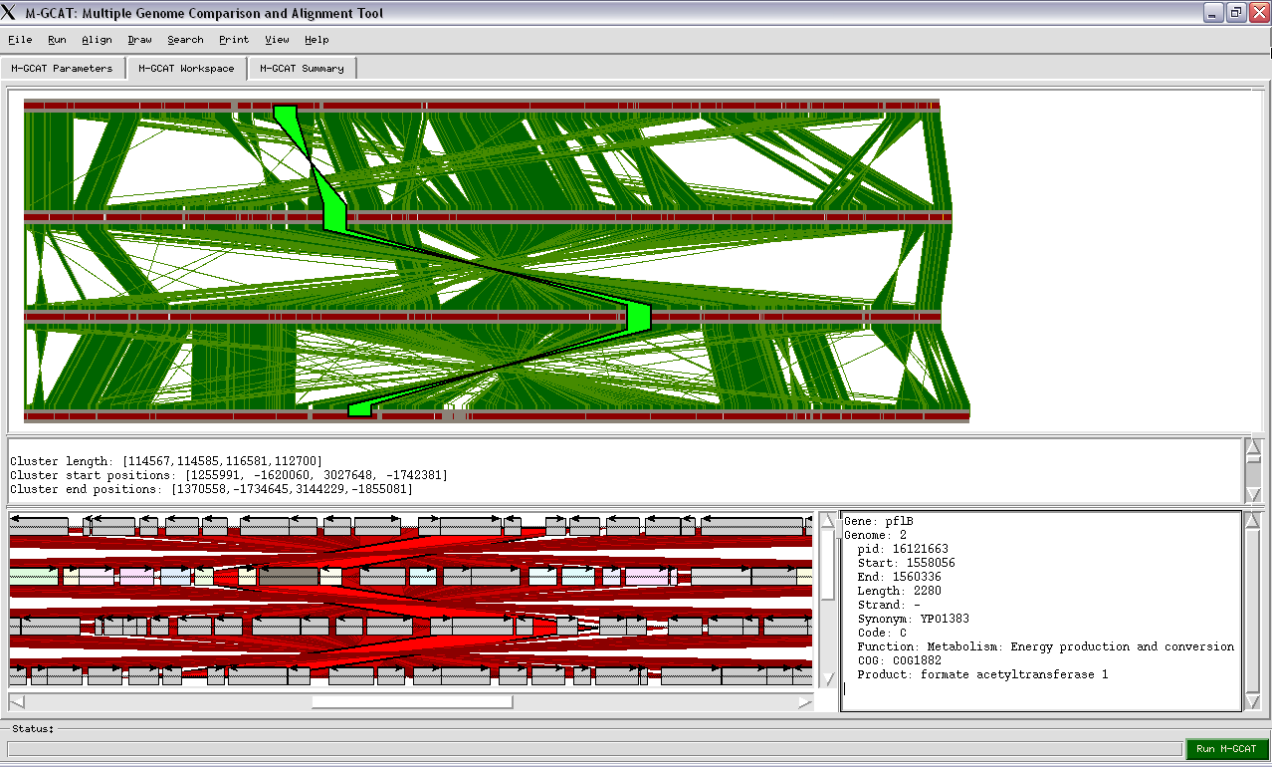
**The M-GCAT genome comparison workspace**. The M-GCAT genome comparison workspace showing the multi-MUMs, multi-MUM clusters, global multiple alignment, gene map, and an orthologous gene between four complete bacterial sequences: *Yersinia pestis biovar Mediaevails*, *Yersinia pestis CO92*, and *Yersinia pseudotuberculosis IP32953*. By analyzing the visual results for this comparison we can quickly observe that these sequences are highly similar, and except for a few smaller regions, there is high sequence identity across all genomes. The green vertical rectangles represent multi-MUM clusters, and the inverted green vertical rectangles indicate regions containing large-scale rearrangements. The highlighted(light green) multi-MUM cluster is an example of a region that was aligned among all genomes. In the gene map window genes are drawn as horizontal rectangles, and all genes annotated in the corresponding PTT file will be displayed. The genes are color coded by function, and a legend is provided at the bottom for quick reference when analyzing the genomes. The vertical lines between the genes represent the multi-MUMs found during comparison.

**Figure 4:**
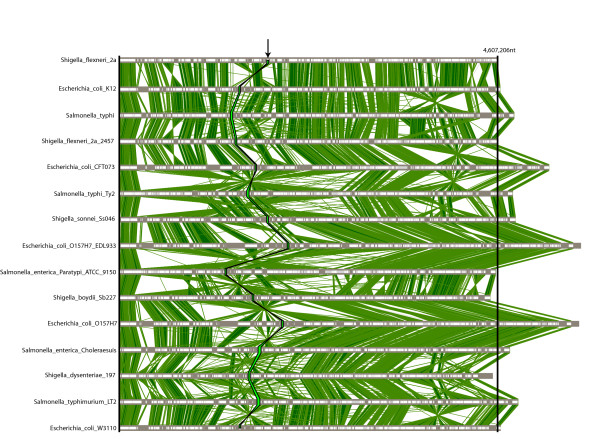
**Multiple genome comparison framework for 15 microbial genomes**. The M-GCAT results of a comparison showing the global alignment framework constructed for the 15 enterobacterial genomes used in sequence set 19. There are 1218 multi-MUM clusters displayed, covering approximately 54.9% of the total genomic sequence. The region highlighted in green and indicated with the black arrow is one of the 1218 regions found to be highly conserved among the 15 closely related species.

**Figure 5:**
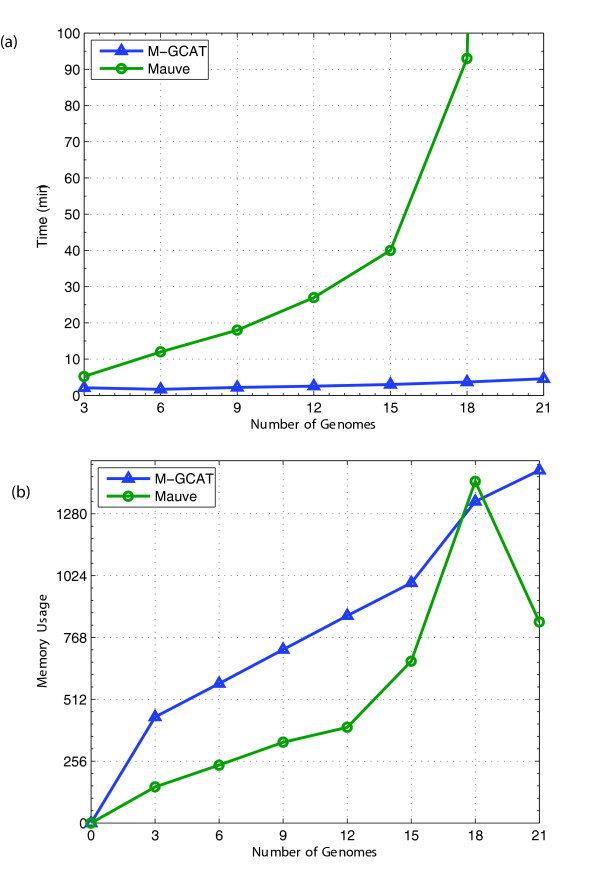
**Analysis of multiple genome comparison framework efficiency and memory usage**. This experiment was ran exclusively on a 2 Ghz Pentium M processor, with 2 GB of main memory, running Windows XP Professional. The memory usage as the peak memory usage during the comparison. The time is represented in total cpu time.

**Table 3:** Multi-MUM search comparison

	M-GCAT		M-GCAT w/Inversions		EMAGEN-DM		MGA	
	*E. Coli*	*Strep*	*E. Coli*	*Strep*	*E. Coli*	*Strep*	*E. Coli*	*Strep*

Running time(s)	32+190	11+26	32+210	11+32	178+223	70+15	535+441	338+382
Number of LIS-MUMs	34844	5568	36154	10238	34612	3781	34922	5503
Total Length of LIS-MUMs	3592285	631663	4012435	1540505	3484053	425309	3547621	626112

Comparison of multi-MUM search efficiency. MGA and EMAGEN-DM results on a Sun Blade 1000 workstation with 1 GB RAM, as reported in [14]. M-GCAT results on a Sun Ultra-250 with a 400 Mhz processor and 512 MB RAM.

**Table 4:** Comparison of M-GCAT & Mauve alignment frameworks.

	M-GCAT		Mauve	
	MUM Clusters	Coverage	LCBs	Coverage

*Entero *3	126	80.1%	126	86.4%
*Entero *6	72	81.0%	91	85.2%
*Entero *9	85	75.5%	113	82.0%

## Supplementary Material

Additional File 3 Configuring M-GCAT Parameters. A detailed description of the main configurable parameters. Click here for file

Additional File 1 90 bacterial genome comparison. Multiple genome comparison framework involving 90 genomes, generated with M-GCAT. Click here for file

Additional File 2 Sequence data. Table of sequences used in all of the experiments. Click here for file
