# Supplementary material for: M-GCAT: interactively and efficiently constructing large-scale multiple genome comparison frameworks in closely related species
Source: BMC Bioinformatics. 2006 Oct 5;7:433. doi: 10.1186/1471-2105-7-433 (PMC1629028; doi:10.1186/1471-2105-7-433)
Supplement: Additional File 3 — Configuring M-GCAT Parameters. A detailed description of the main configurable parameters. [file 1471-2105-7-433-S3.pdf]

Category	Name	Description
MUM		
	**Min Anchor length**	Min Anchor length is the minimum allowable size for the initial set of multi-MUM anchors found among all genomes to establish the framework for the global alignment. The default value is $\log_2(\text{length}(S_1))$, where S_1 is the reference sequence.
	**Min MUM length**	This is the minimum allowable length for new multi-MUMs found between the anchors during the recursive anchoring process. As searchable sequence regions between the initial multi-MUM anchors become smaller and smaller, so should this value. The default value is $1.3 * \log_2(\text{length}(R_n))$, where R_n is a searchable sequence region in sequence n and 1.3 is a coefficient that should be decreased when dealing with more distantly related species.
	**Random MUM length**	As it can quickly become difficult to distinguish spurious matches from meaningful homology, we provide the user with a parameter that estimates a statistically significant match size for the given sequence comparison. All multi-MUMs less than or equal to this length that are also break-points in conserved sequence collinearity are considered to be random will be removed in the Filtering process. The default value is 0, but it can be adjusted depending on the length and the number of the input genomes.
CLUSTER		
	**Q**	This value is the minimum allowable length in nucleotides of a searchable sequence region R_n where M-GCAT will perform a search for new multi-MUMs. Decreasing this value will usually generate more multi-MUMs and decrease alignment time, while increasing the recursive anchoring time. The default value is 100nt.
	**D**	This is the maximum allowable distance, in nucleotides, between any two non-overlapping adjacent multi-MUMs in a cluster. Increasing this value will generally increase the alignment time, and decreasing this value will generally decrease the percentage of the genomes that will be aligned. The default value is 1000nt.
	**P**	The Partition (P) value is used to partition large genomes into several smaller parts in order to reduce memory usage. The default value is 10,000,000 nt. A comparison involving a sequence of 20,000,000 nt would then require approximately *50%* less memory, but would imply a corresponding increase in runtime.
