# Supplementary material for: M-GCAT: interactively and efficiently constructing large-scale multiple genome comparison frameworks in closely related species
Source: BMC Bioinformatics. 2006 Oct 5;7:433. doi: 10.1186/1471-2105-7-433 (PMC1629028; doi:10.1186/1471-2105-7-433)
Supplement: Additional File 2 — Sequence data. Table of sequences used in all of the experiments. [file 1471-2105-7-433-S2.pdf]

#	Sequence Name	NCBI Accession
1	*Mycoplasma pneumoniae*	NC_000912.1
	Mycoplasma genitalium	NC_000908.1
2	*Pyrococcus horikoshi*	NC_000961.1
	Pyrococcus abyssi	NC_000868.1
3	*Salmonella enterica subsp. enterica serovar Typhi str. CT18*	NC_003198.1
	Salmonella enterica subsp. enterica serovar Typhi Ty2	NC_004631.1
4	*Listeria monocytogenes str. 4b F2365*	NC_002973.6
	Listeria innocua Clip11262	NC_003212.1
	Listeria monocytogenes EGD-e	NC_003210.1
5	*Xanthomonas campestris pv. campestris str. ATCC 33913*	NC_003902.1
	Xanthomonas campestris pv. campestris str. 8004	NC_007086.1
	Xanthomonas campestris pv. vesicatoria str. 85-10	NC_007508.1
6	*Pseudomonas syringae pv. phaseolicola 1448A*	NC_005773.3
	Pseudomonas syringae pv. tomato str. DC3000	NC_004578.1
	Pseudomonas syringae pv. syringae B728a	NC_007005.1
7	*Chlamydophila pneumoniae AR39*	NC_002179.2
	Chlamydophila pneumoniae CWL029	NC_000922.1
	Chlamydophila pneumoniae J138	NC_002491.1
	Chlamydophila pneumoniae TW-183	NC_005043.1
8	*Yersinia pestis biovar Medievalis str. 91001*	NC_005810.1
	Yersinia pestis CO92	NC_003143.1
	Yersinia pestis KIM	NC_004088.1
	Yersinia pseudotuberculosis IP 32953	NC_006155.1
9	*Shigella sonnei Ss046*	NC_007384.1
	Shigella boydii Sb227	NC_007613.1
	Shigella dysenteriae 197	NC_007606.1
	Shigella flexneri 2a str. 2457T	NC_004741.1
	Shigella flexneri 2a str. 301	NC_004337.1
10	*Salmonella typhimurium LT2*	NC_003197.1
	Salmonella enterica subsp. enterica serovar Choleraesuis str. SC-B67	NC_006905.1
	Salmonella enterica subsp. enterica serovar Paratyphi A str. ATCC 9150	NC_006511.1
	Salmonella enterica subsp. enterica serovar Typhi str. CT18	NC_003198.1
	Salmonella enterica subsp. enterica serovar Typhi Ty2	NC_004631.1
11	*Escherichia coli W3110 DNA*	AC_000091.1
	Escherichia coli O157:H7	NC_002695.1
	Escherichia coli CFT073	NC_004431.1
	Escherichia coli K12	NC_000913.2
	Escherichia coli O157:H7 EDL933	NC_002655.1
12	*Streptococcus pyogenes SSI-1*	NC_004606.1
	Streptococcus pyogenes M1 GAS	NC_002737.1
	Streptococcus pyogenes MGAS315	NC_004070.1
	Streptococcus pyogenes MGAS5005	NC_007297.1
	Streptococcus pyogenes MGAS6180	NC_007296.1
	Streptococcus pyogenes MGAS8232	NC_003485.1
	Streptococcus pyogenes MGAS10394	NC_006086.1
13	*Staphylococcus aureus subsp. aureus USA300*	NC_007793.1
	Staphylococcus aureus subsp. aureus COL	NC_002951.2
	Staphylococcus aureus subsp. aureus Mu50	NC_002758.2
	Staphylococcus aureus subsp. aureus MW2	NC_003923.1
	Staphylococcus aureus subsp. aureus N315	NC_007795.1
	Staphylococcus aureus subsp. aureus NCTC 8325	NC_007622.1
	Staphylococcus aureus RF122	NC_006155.1
14	*Bacillus anthracis str. 'Ames Ancestor'*	NC_007530.2
	Bacillus anthracis str. Ames	NC_003997.3
	Bacillus anthracis str. Sterne	NC_005945.1
	Bacillus cereus ATCC 14579	NC_004722.1
	Bacillus cereus ATCC 10987	NC_003909.8
	Bacillus cereus ZK	NC_006274.1
	Bacillus thuringiensis serovar konkukian str. 97-27	NC_005957.1
